# Influence of music on steroid hormones and the relationship between receptor polymorphisms and musical ability: a pilot study

**DOI:** 10.3389/fpsyg.2013.00910

**Published:** 2013-12-03

**Authors:** Hajime Fukui, Kumiko Toyoshima

**Affiliations:** Department of Education, Nara University of EducationNara, Japan

**Keywords:** music, cortisol, testosterone, 17-beta estradiol, androgen receptor, polymorphism, AMMA

## Abstract

Studies have shown that music confers plasticity to the brain. In a preliminary pilot study, we examined the effect of music listening on steroid hormones and the relationship between steroid hormone receptor polymorphisms and musical ability. Twenty-one subjects (10 males and 11 females) were recruited and divided into musically talented and control groups. The subjects selected (1) music they preferred (chill-inducing music) and (2) music they did not like. Before and after the experiments, saliva was collected to measure the levels of steroid hormones such as testosterone, estradiol, and cortisol. DNA was also isolated from the saliva samples to determine the androgen receptor (AR) and arginine vasopressin receptor 1A genotypes. Advanced Measures of Music Audiation (AMMA) was used to determine the musical ability of the subjects. With both types of music, the cortisol levels decreased significantly in both sexes. The testosterone (T) levels declined in males when they listened to both types of music. In females, the T levels increased in those listening to chill-inducing music but declined when they listened to music they disliked. However, these differences were not significant. The 17-beta estradiol levels increased in males with both types of music, whereas the levels increased with chill-inducing music but declined with disliked music in females. The AMMA scores were higher for the short repeat length-type AR than for the long repeat length-type. Comparisons of AR polymorphisms and T levels before the experiments showed that the T levels were within the low range in the short repeat length-type group and there was a positive relationship with the repeat length, although it was not significant. This is the first study conducted in humans to analyze the relationships between the AR gene, T levels, and musical ability.

## Introduction

Significant progress has been made in understanding the roles of psychophysiological processes in musical emotions during recent years (Hodges, 2010). Accumulating evidence demonstrates that listening to music can elicit pronounced physiological and emotional responses (Lewis, 2002). Studies investigated the effects of music perception and production on the autonomic nervous system (and consequently on hormones) as well as on the immune system. Although there is a recognition of the effects of music on the immune system (including neurotransmitters and hormones) and human health, very little is known about the cognitive influences and the underlying neural correlates of these effects. Research in this area could also provide explanations of the physiological effects of music (Koelsch and Siebel, 2005).

It is believed that music confers neuronal plasticity and is involved in the learning process and readjustment. An example is the response of brain cells to musical stimuli. This effect is believed to be persistent, although the precise mechanism remains unknown (Wan and Schlaug, 2010; Moreno et al., 2011; Trainor et al., 2012).

Steroid hormones may hold the key to unlocking the mechanism that underlies the effect of music on neurons because they confer neuronal plasticity. In particular, T and estrogen are deeply involved in brain cell regeneration, restoration, and protection. They also have strong connections with recognition, memory, and emotion and thus may be associated with mental disorders (Fukui and Toyoshima, 2008). Previous studies demonstrated a gender-specific relationship between steroid hormones and musical ability (Hassler et al., 1985; Hassler, 1991; Fukui and Yamashita, 2003). In the field of behavioral endocrinology and neuroendocrinology, many studies have shown that musical stimulation (listening) affects various biochemical substances (Hassler et al., 1992; VanderArk and Ely, 1993; Kreutz et al., 2004; for reviews, see Hodges, 2010; Chanda and Levitin, 2013). In particular, many studies have shown that listening to music is effective for alleviating and relieving stress. Stress reduction due to music listening and performance has been attributed to reductions in cortisol (C) levels (Khalfa et al., 2003; Nater et al., 2006; Toyoshima et al., 2011). Listening to music also changes T levels (increases and decreases) (Fukui, 2001; Fukui and Yamashita, 2003). Studies have shown that musical activities (listening and playing) affect steroid secretion in elderly individuals and are likely to alleviate psychological states such as anxiety and tension. Furthermore, steroid levels may vary in both directions, i.e., increasing in subjects with low hormone levels and decreasing in subjects with high hormone levels (Fukui and Toyoshima, 2008; Fukui et al., 2012). It is plausible that music confers neuronal plasticity by affecting steroid hormones, irrespective of whether the mechanisms are genomic or non-genomic.

Recent studies have shown that there is a substantial genetic component to music perception, including absolute pitch (Theusch et al., 2009), congenital amusia (Peretz et al., 2007), auditory structuring ability (Ukkola et al., 2009), and musical ability (Peretz, 2006; Morley et al., 2012; Park et al., 2012; Kanduri et al., 2013; Ukkola-Vuoti et al., 2013).

In this preliminary pilot study, we examined the effect of listening to chill-inducing music on the levels of steroid hormones, i.e., C, T, and 17-beta estradiol (E), and the relationship between the hormone androgen receptor (AR) and arginine vasopressin receptor 1A (AVPR1A) polymorphisms, and musical ability. We investigated AR because the biological actions of T are mediated by intracellular receptors that exert transcriptional control over androgen-dependent genes, which are expressed in various brain regions (Manuck et al., 2010). In addition, cell membranes possess ARs and cellular responses to these receptors are much faster than those to intracellular receptors (Heinlein and Chang, 2002). Many biological actions of T and other androgens are mediated by ARs, which are expressed in diverse areas of the brain (Gatchel and Zoghbi, 2005).

The transactivation potential of AR appears to decline with increasing numbers of CAG repeats. These repeats are normally distributed over a range of 11–37 and usually average 21–22 in Caucasian populations (Tut et al., 1997; Edwards et al., 1999; Platz et al., 2000). Moreover, AR polymorphisms appear to be associated with sociality, aggressiveness, and depression or autism (Henningsson et al., 2009; Vermeersch et al., 2010; Sankar and Hampson, 2012). The relationship between AR polymorphisms and behavioral phenotypes remains to be elucidated, but some evidence suggests that a high number of CAG repeats is associated with cognitive aging (Yaffe et al., 2003), whereas a low number of repeats is associated with violence and aggressiveness, as well as depressive symptomatology and autism, in correlation with basal T levels (Jönsson et al., 2001; Seidman et al., 2001; Cheng et al., 2006; Rajender et al., 2008; Manuck et al., 2010).

Several studies investigated the development of sexually dimorphic AVP systems in mammals and other vertebrates and the role played by gonadal hormones in their regulation (for a review, see De Vries and Panzica, 2006). It is hypothesized that androgens, via the actions of their receptors, should have roles in the organization and modulation of the AVP parvocellular sexually dimorphic system (Allieri et al., 2013).

The relationship between AVPR and sociality has been described in several reports (Ebstein et al., 2010). Recent studies demonstrated associations between microsatellites (RS1 and RS3) in the promoter region of AVPR1A and autistic disorder (Wassink et al., 2004) and social behavior (Bachner-Melman et al., 2005; Yirmiya et al., 2006). The AVP level has been implicated in aggression (Coccaro et al., 1998) where functional variation in the corresponding receptors may have important roles. The receptor gene AVPR1A contains promoter repeats, which are associated with the regulation of brain expression patterns in lower vertebrates. AVPR1A has been implicated in aggressive behavior in rodents (Ferris et al., 1997).

In mammals, including humans, AVP has a prominent role in controlling higher cognitive functions, such as memory and learning, as well as social, emotional, and behavioral traits, including pair bonding and aggression in males, love, and altruism (Ukkola et al., 2009). A direct relationship has not been confirmed in humans, but it has been proposed that polymorphisms of the two microsatellites are associated with social communication capacity or autism (Kim et al., 2002; Wassink et al., 2004; Bachner-Melman et al., 2005; Craig and Halton, 2009; Ebstein et al., 2010). In particular, the short allele of RS1 reduced the transcription of AVPR1A, which elevates amygdala activity, thereby leading to social withdrawal that characterizes autism (Meyer-Lindenberg et al., 2009; Tansey et al., 2011).

An association has been reported between AVP/AVPR and human musical ability (Bachner-Melman et al., 2005; Ukkola et al., 2009; Ukkola-Vuoti et al., 2011; Dai et al., 2012; Ebstein et al., 2012; Morley et al., 2012). A study of the relationship between musical memory and AVPR1A detected highly significant gene × gene epistatic interactions with promoter region polymorphisms (Granot et al., 2007). A study of the AVPR1A gene (RS1 and RS3), musical aptitude (Karma Music test), Carl Seashore's tests for pitch, and combined music test scores detected an overall haplotype association (Ukkola et al., 2009). In another study that investigated the relationship between music listening, music aptitude (Karma Music test), and AVPR1A polymorphisms (RS3, RS1, and AVR), the willingness to listen to music was associated with neurobiological pathways related to social affiliation and communication (Ukkola-Vuoti et al., 2011).

T and AVP are associated with sociality, i.e., AVP with prosociality and T with antisociality. Thus, studies of the relationship between musical aptitude, musical function, and musical evolution need to consider both receptors (AR and AVPR1A).

## Methods

### Subjects

Twenty-one subjects were enrolled in the study. They were divided into two groups: musically talented and control groups. The musically talented group included professional musicians, top performers from a music college, and subjects who obtained excellent results in a music competition (4 males and 5 females; age: 15–54 years, average age, 35.3 years). The control group did not meet the above recruitment criteria (6 males and 6 females; age: 17–55 years, average age, 35.8 years).

Musical emotions are elicited by complex interactions between the music, the listener, and the environment, which are affected by factors such as personality and culture (Sloboda and Juslin, 2010). Various methods have been used to investigate musical emotions, but a highly reliable approach is focused on the concept of “chills,” which are used by many researchers as subjective indicators of musical feeling (Hodges, 2010; Nusbaum and Silvia, 2010; Balteş et al., 2011). “Musical chills” are a phenomenon that involves strong affective changes such as crying, shivers down the spine, and goose bumps (Gabrielsson, 2001; Gabrielsson and Lindström, 2003). However, not everyone experiences musical chills (Salimpoor et al., 2009). Our aim was to investigate the effects of musical emotions on hormones and their relationships with genes. Therefore, we recruited individuals who had experienced “chills” as an intensely pleasurable response to music (Salimpoor et al., 2009).

### Procedure

Individual subjects listened to music in a quiet room between 13:00 and 18:00 h to control for circadian variations in their hormone levels. The hormone levels are most stable in the afternoon and early evening. Thus, these are the times recommended for studies focusing on individual differences in the hormone levels (Gupta et al., 2000; Fukui and Yamashita, 2003; Yang et al., 2007).

We decided to collect saliva samples for this study because this was less invasive and stressful than drawing blood. The saliva hormone levels are highly correlated with the serum hormone levels and are representative of the free and biologically active steroid fraction (Poll et al., 2007). Each subject provided 2 ml of saliva at the beginning of the experiment to determine their baseline levels.

DNA used to determine the AR and AVPR1A (RS1) genotypes was isolated from approximately 2 ml saliva samples, which were collected in sterile containers.

### Music

For this experiment, we asked subjects to choose a musical piece from two types of music: (1) music they preferred (chill-inducing music) and (2) music they did not like.

The two stimulus categories were each presented for 5 min. The subjects who selected music that lasted for >5 min were asked to choose a 5-min section that contained the most chill-inducing or most disliked part within the same piece. The preferred music selected by the subjects (chill-inducing) included classical piano and orchestral music, Japanese pop, and wind music. The disliked music included contemporary music, folk songs, and video game music. Most of the musical pieces were instrumental music.

### Musical ability

We used the melodic imagery subtest from Advanced Measures of Music Audiation (AMMA: GIA Publications, Inc.) by Edwin E. Gordon (Gordon, 1965), which requires subjects to judge whether a response to music is a variant of a reference music phrase and to compare pairs of melodic phrases where the melodic information is retained but the rhythm may change.

AMMA is a valid musical aptitude test for college students (music majors and non-majors), high school students, and junior high students. The entire test takes <20 min to complete and yields tonal, rhythm, and composite scores.

### Hormone measurement

Each subject provided a 2 ml saliva sample before and after listening to music. The samples were immediately frozen at −20°C after collection, and C, T, and E levels were measured by luminescence-based immunoassay (FilterMax F3 Austria; IBL Germany). The inter- and intra-assay coefficients of variation were 2.1 and 3.4% for C, 6.96 and 1.47% for T, and 11.9 and 7.2% for E, respectively.

### Genotyping

To minimize saliva impurities, the subjects abstained from eating, drinking fluids other than water, smoking, chewing gum, or brushing their teeth for 30 min before sample collection.

Approximately 2 ml of saliva was collected from the subjects in a sterile Oragene DNA vial (DNA Genotek, Inc., Kanata, Ontario, Canada). After collection, the entire saliva sample (2 ml) was mixed with 2 ml of Oragene DNA-stabilizing solution. Saliva sampling produces a higher DNA yield than saliva obtained from mouthwash or buccal swabs, and quality of DNA obtained is better than that of DNA from buccal swabs. The median yield using the Oragene method is 110 μg (Sankar and Hampson, 2012).

Using a 50 ng DNA extract, the CAG repeat region of the AR gene was amplified by polymerase chain reaction: forward primer, (fluorescent), 5′-CTTTCCAGAATCTGTTCCAG-3′; reverse primer, 5′-GAAGGTTGCTGTTCCTCATC-3′.

The sample was heated at 95°C for 3 min, followed by 35 cycles at 95°C for 1 min, 55°C for 1 min, and 72°C for 1 min, with a final incubation at 72°C for 10 min. Repeat numbers (CAGn) were confirmed by sequencing a reference subset of samples with alleles of different lengths.

Using a 50 ng DNA extract, RS1 (GATA) microsatellites were amplified using the following primers, as described by Thibonnier et al. (2000) (corresponding to the GATA microsatellite at position 553): forward primer (fluorescent), 5′-AGG GAC TGG TTC TAC AAT CTG C- 3′; reverse primer, 5′-ACC TCT CAA GTT ATG TTG GTG G-3′. Each reaction mixture contained 0.5 mM primer. The sample was heated at 95°C for 5 min, followed by 30 cycles at 95°C for 30 s, 55°C for 30 s, 72°C for 40 s, and a final extension step at 72°C for 10 min. Nine alleles (302–334) were identified for RS1, and the allele frequency distribution was similar to that in previous reports (Yirmiya et al., 2006).

The amplified fragments were subjected to capillary electrophoresis and read using CEQ8800 (Beckman, USA). The lengths of the repeat regions in each sample were quantified using GenomeLab™ GeXP Advance.

## Results

**Figure 1:**
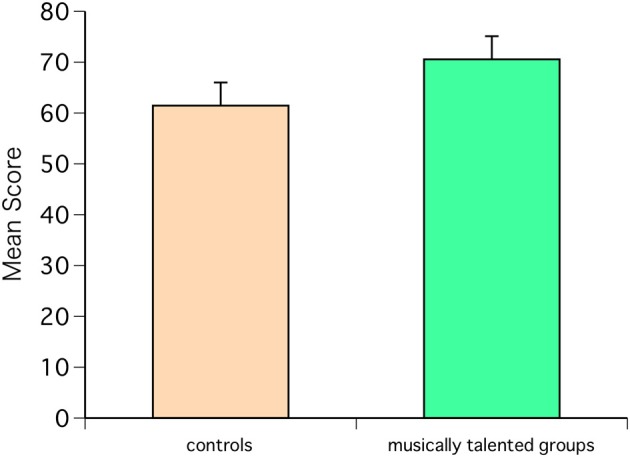
**Advanced Measures of Music Audiation (AMMA) scores for the musically talented and control groups**. The scores of the musically talented group were higher than those of the control group [*F*_(1, 18)_ = 8.86, *P* = 0.0081].

One subject was excluded because the responses to AMMA were incomplete. AMMA showed that the scores of the musically talented group were significantly higher than those of the control group [*F*_(1, 18)_ = 8.86, *P* = 0.0081] (Figure 1).

**Figure 2:**
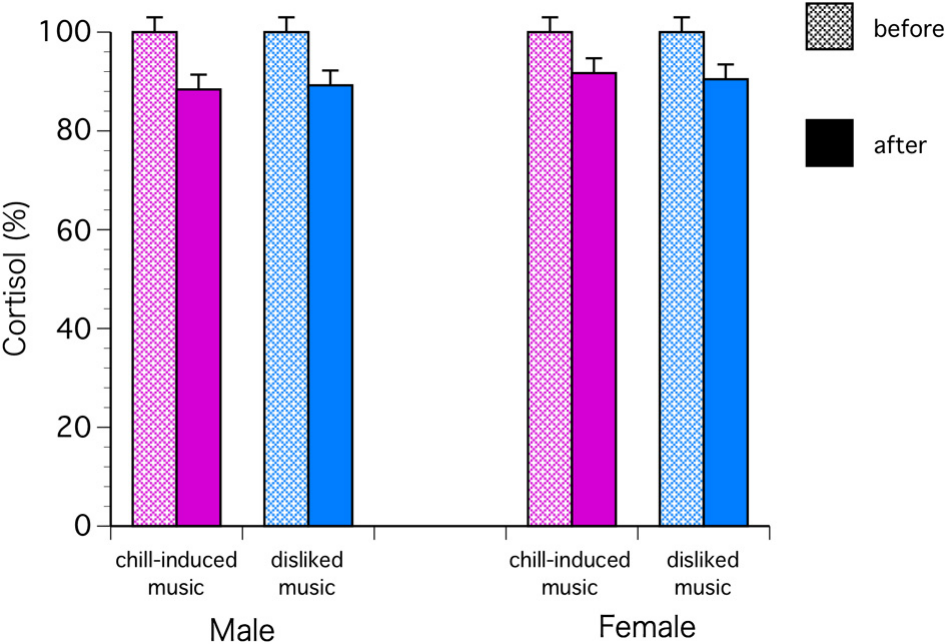
**The cortisol levels decreased significantly in both sexes with chill-inducing and disliked music [*F*_(1, 68)_ = 9.57, *P* = 0.0029; Bonferroni *P* < 0.0001]**.

**Figure 3:**
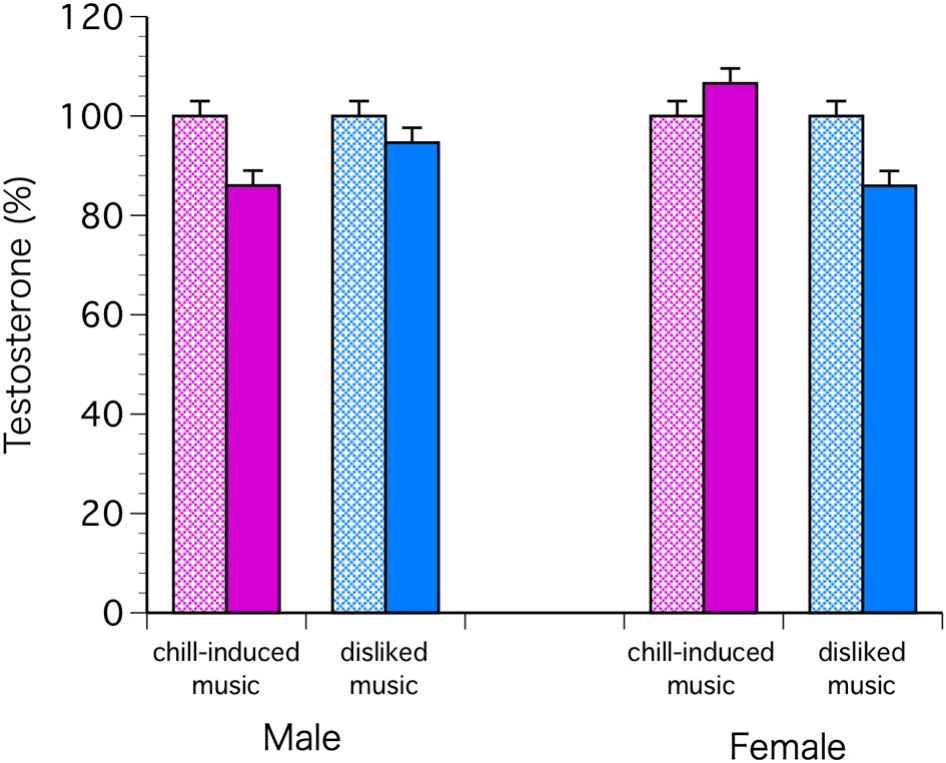
**The T levels decreased in males who listened to both types of music**. In females, the T levels increased in those who listened to chill-inducing music but decreased in those who listened to disliked music. However, there was no significant interaction between changes in T levels, sex, two stimuli, and the groups (musically talented and control) [*F*_(1, 68)_ = 4.76, Bonferroni *P* = 1.0].

**Figure 4:**
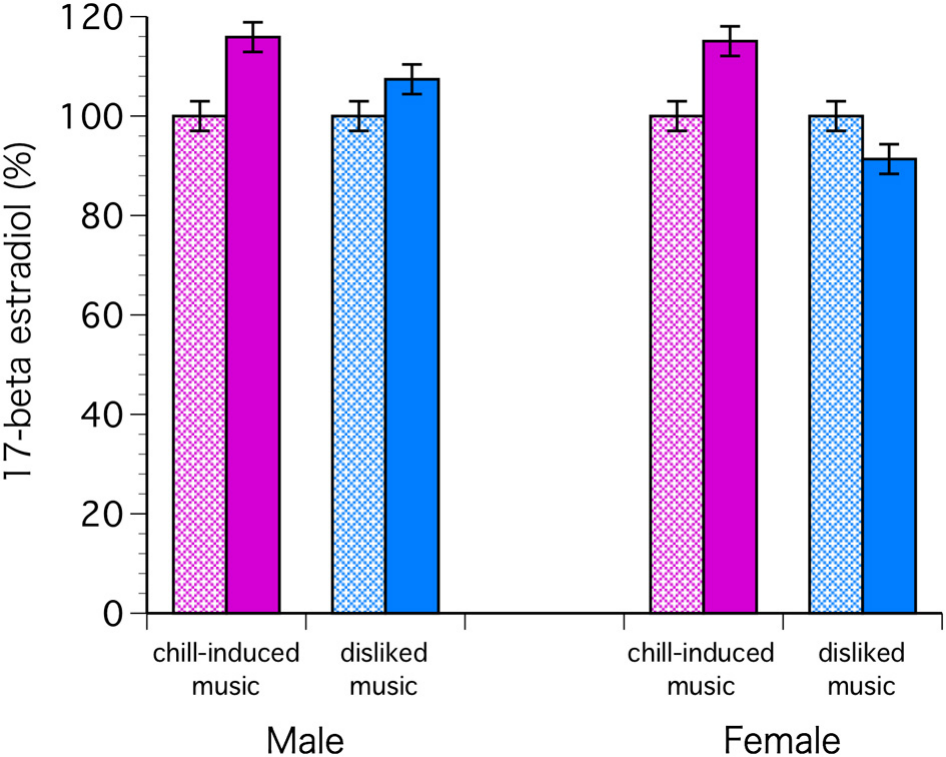
**ANOVA (E change × sex × two stimuli × groups) showed that the E levels changed significantly before and after the stimuli [*F*_(1, 68)_ = 4.93, *P* = 0.0297; Bonferroni *P* < 0.0001], and a significant difference was observed between the two stimuli [*F*_(1, 68)_ = 5.62, *P* = 0.0206; Bonferroni *P* < 0.0001]**. The E levels increased in males who listened to both types of music. In females, the E levels increased with chill-inducing music but decreased with disliked music.

The C levels decreased significantly in both sexes with chill-inducing and disliked music [*F*_(1, 68)_ = 9.57, *P* = 0.0029; Bonferroni *P* < 0.0001] (Figure 2). The T levels decreased in males who listened to both types of music. In females, the T levels increased in those who listened to chill-inducing music but decreased in those who listened to disliked music (Figure 3). However, there was no significant interaction between changes in T levels, sex, two stimuli, and the groups (musically talented and control) [*F*_(1, 68)_ = 4.76, Bonferroni *P* = 1.0]. ANOVA (E change × sex × two stimuli × groups) showed that the E levels changed significantly before and after the stimuli [*F*_(1, 68)_ = 4.93, *P* = 0.0297; Bonferroni *P* < 0.0001], and a significant difference was observed between the two stimuli [*F*_(1, 68)_ = 5.62, *P* = 0.0206; Bonferroni *P* < 0.0001]. The E levels increased in males who listened to both types of music. In females, the E levels increased with chill-inducing music but decreased with disliked music (Figure 4). However, no significant interaction was observed between the changes in E levels and the two stimuli [*F*_(1, 68)_ = 5.62, *P* = 0.0206; Bonferroni *P* = 0.7602]. These changes did not differ between the musically talented and control groups [C: *F*_(1, 68)_ = 0.52, *P* = 0.4736; T: *F*_(1, 68)_ = 0.22, *P* = 0.6417; E: *F*_(1, 68)_ = 0.96, *P* = 0.3318].

The mean value was calculated for each hormone before the stimulus, and the relationship with the AMMA scores was analyzed. No relationships were observed between the E and C levels and the AMMA scores in either sex. In females, however, the T levels produced an inverted U-shaped graph when they were plotted against the test results.

**Figure 5:**
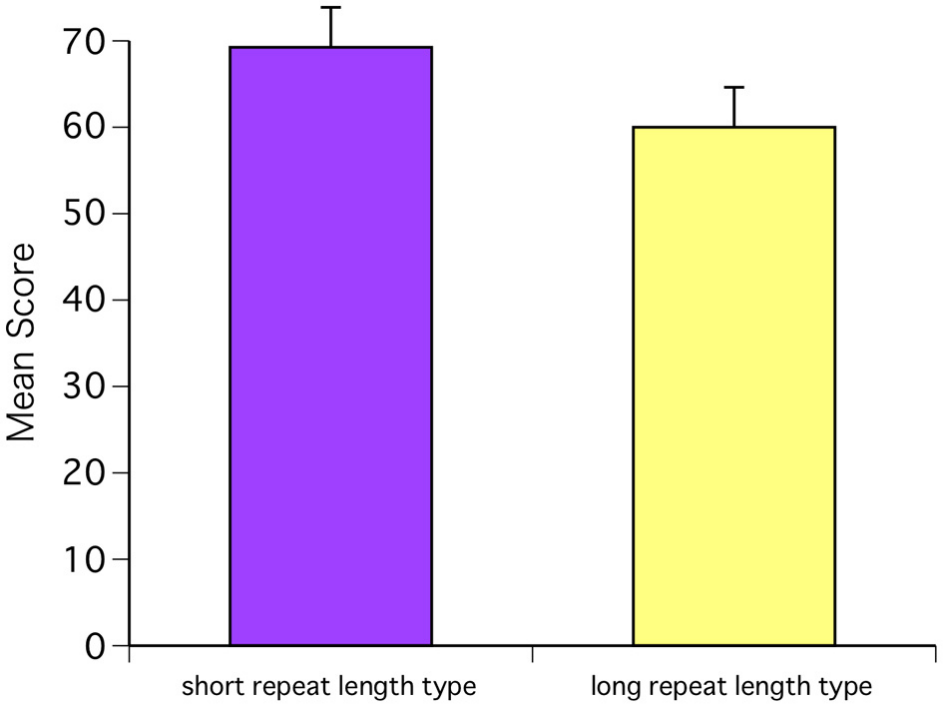
**The CAG repeat length in ARs ranged from 3 to 20 among the study subjects, with a median of 7**. In both sexes, higher test scores were observed in the subjects with a short repeat length (<7 repeats, *N* = 12: 4 males and 8 females) than in those with a long repeat length (>7 repeats, *N* = 8: 5 males and 3 females) [*F*_(1, 18)_ = 8.88, *P* = 0.0080; Bonferroni *P* = 0.0001].

Next, we investigated the relationship between the AMMA scores and receptor polymorphisms. One subject was excluded because the repeats could not be specified. The CAG repeat length in ARs ranged from 3 to 20 among the study subjects, with a median of 7. In both sexes, higher test scores were observed in the subjects with a short repeat length (<7 repeats, *N* = 12: 4 males and 8 females) than in those with a long repeat length (>7 repeats, *N* = 8: 5 males and 3 females) [*F*_(1, 18)_ = 8.88, *P* = 0.0080; Bonferroni *P* = 0.0001] (Figure 5). Next, we compared AR polymorphisms and baseline T levels for each hormone. One subject was excluded because hormone levels could not be measured. Baseline T levels for each group indicated that the T levels in the short repeat length-type group were within the low range and showed a positive relationship with the repeat length. However, these differences were not significant [*F*_(1, 17)_ = 1.06, *P* = 0.3167]. There were no differences between AR polymorphism and baseline C levels [*F*_(1, 17)_ = 0.28, *P* = 0.6045] and neither AR polymorphism and baseline E levels [*F*_(1, 17)_ = 0.48, *P* = 0.4996].

The allele and genotype distributions of AVPR polymorphisms (RS1) were similar to those reported in previous studies (Kim et al., 2002; Meyer-Lindenberg et al., 2009).

The AMMA scores were higher for the short repeat length-type AVPR1A (RS1) (<310-bp repeats, *N* = 12: 4 males and 8 females) than for the long repeat length-type (>314-bp repeats, *N* = 8: 5 males and 3 females), although the differences were not significant [*F*_(1, 18)_ = 0.75, *P* = 0.3994]. No relationship was found between AVPR1A (RS1) and any of the hormones tested [C: *F*_(1, 17)_ = 0.83, *P* = 0.3736; T: *F*_(1, 17)_ = 0.03, *P* = 0.8706; E: *F*_(1, 17)_ = 0.64, *P* = 0.4337].

We investigated the relationships between the receptors and the changes in each hormone. However, these differences were not significant in AR [C: *F*_(1, 64)_ = 0.50, *P* = 0.4841; T: *F*_(1, 64)_ = 0.74, *P* = 0.3924; E: *F*_(1, 64)_ = 0.05, *P* = 0.8166] and AVPR (RS1) [C: *F*_(1, 64)_ = 0.50, *P* = 0.4800; T: *F*_(1, 64)_ = 1.54, *P* = 0.2190; E: *F*_(1, 64)_ = 0.04, *P* = 0.8518].

## Discussion

Several studies reported a relationship between C levels and musical appreciation. In general, these studies have shown that listening to music lowers the C levels, although there are slight differences, depending on the preferred types or genre of music. Differences in the C levels are not gender-specific (Fukui and Yamashita, 2003; Ventura et al., 2012). In general, both sexes enjoy listening to chill-inducing music. However, we also observed that the C levels decreased in subjects who listened to music they disliked, which differs from the results of previous studies, including our own research (for a review, see Hodges, 2010). Listening to music that one dislikes is usually a stress inducer, which can lead to elevated C levels. One study showed that no mood induction procedures were associated with changes in the C levels (Hucklebridge et al., 2000). Further research is required to determine whether the use of chill-inducing music caused this discrepancy.

Although it was not significant, T levels decreased in males with chill-inducing and disliked music. Very few studies investigated the effects of music appreciation on the T levels (Fukui, 2001; Fukui and Yamashita, 2003). Previous studies have shown that there is a gender-specific difference in the change in T levels when listening to music. For example, in adolescents and men in the late middle age, the T levels decreased with music they liked, whereas the levels increased with music they disliked. In contrast, the T levels were elevated in females, irrespective of the type of music (Fukui, 2001; Fukui and Yamashita, 2003). However, the elevated T levels were also found during dance, which suggests that there is a relationship between music and movement (Murcia et al., 2009). Our results showed that music reduced the T levels in the male subjects, irrespective of the type of music. In the female subjects, the T levels increased with chill-inducing music whereas the levels decreased with disliked music. These results differ from the results of our previous study of adolescents and late middle-aged subjects (Fukui, 2001; Fukui and Yamashita, 2003).

The E levels significantly increased in males with chill-inducing and disliked music. In females, the E levels increased with chill-inducing music but decreased with disliked music. Studies of the E levels are rare. We previously conducted the only study, to the best of our knowledge, to use the E levels as an indicator, where we investigated the effects of singing in a choir for 90 min in elderly female subjects (average age, 72.9 years) on hormone levels, rather than listening to music. The T levels were also measured in that study, which showed that changes in the hormone levels depended on their baseline levels. Indeed, the hormone levels increased in the subjects with low baseline levels after musical activity, whereas the levels decreased in those with high baseline levels. We named this phenomenon the adjusting effect (Fukui and Toyoshima, 2011). In the present study, we did not find an adjusting effect. Thus, future studies should investigate whether the T and E levels are adjusted in response to listening to music in adolescent and late middle-aged subjects.

We examined the relationships between the hormone levels and AMMA scores and found that plotting the T levels against the AMMA scores produced an inverted U-shaped graph in females. A gender-specific relationship between music aptitude and the T levels has been reported previously (Kemp, 1985; Gouchie and Kimura, 1991; Hassler, 1991). In general, this relationship was positive in females and negative in males, which indicates that men with low T levels and women with high T levels are more musically talented. Although a difference was observed in the T levels between males and females, there was a continuum of values, where some women had levels as high as those observed in some men. Borniger et al. (2013) reported that female music students tended to have higher T levels than female non-music students, but no significant relationship was observed between the T levels and AMMA scores of males or females. In the present study, females with very high or very low T levels scored poorly in the test, whereas those with moderately high T levels scored very well. These results support the results of previous studies, which suggest the existence of optimal T levels for music aptitude (Hassler, 1991; Fukui, 2001).

Intelligence is considered one of the factors that affected test results because of the link between genotype and cognitive ability (Spearman, 1904; Jensen, 1993; Deary et al., 2010). Individual differences in human intelligence are associated with genetic variation (Davies et al., 2011). In particular, the large number of X chromosome-linked mental retardation syndromes indicates an association between the X chromosome and mental impairment. Thus, the X chromosome is assumed to have a strong effect on human intelligence (Zechner et al., 2001; Skuse, 2005). A previous study reported that the Japanese population had comparatively shorter CAGs than the Caucasian population, although the difference was not significant (Sasaki et al., 2003). We considered that our samples were not exceptional.

Our results showed that the AMMA scores of the subjects with short repeat lengths were significantly higher in both sexes. Previous studies have shown that AR polymorphisms (the number of CAG repeats) are inversely correlated with receptor function, which reflects the T levels (Zitzmann, 2009; Manuck et al., 2010; Sankar and Hampson, 2012). Thus, the T levels are expected to be high if the repeat sequences are short, and vice versa. We also detected a positive relationship between the T levels (baseline values) and AR polymorphisms (repeat length), although it was not significant. Our results support earlier studies, which demonstrated a relationship between low T levels and musical aptitude, but they differ from other findings that short repeat sequences reflect high T levels. It is unclear whether this difference is due to some defect in ARs of the musically talented group or whether there is some other underlying cause. Our study was only a pilot study, and further studies need to be conducted in a larger population.

The test results for the subjects with short AVPR repeat sequences were higher than for those with long repeat sequences, although the differences were not significant. AVPR is associated with sociality, where the short sequence type is linked to antisocial and autistic behavior. An inverse relationship between musical talent and sociality has also been reported (Allen et al., 2009). Previous results are very interesting but no firm conclusions can be drawn, which means that further studies are essential.

### Conflict of interest statement

The authors declare that the research was conducted in the absence of any commercial or financial relationships that could be construed as a potential conflict of interest.
